# In-vitro model systems to study Hepatitis C Virus

**DOI:** 10.1186/1479-0556-9-7

**Published:** 2011-04-06

**Authors:** Usman Ali Ashfaq, Shaheen N Khan, Zafar Nawaz, Sheikh Riazuddin

**Affiliations:** 1 Division of Molecular Medicine, National Centre of Excellence in Molecular Biology, University of the Punjab, Lahore, Pakistan; 2 Braman Family Breast Cancer Institute, University of Miami, USA; 3 Allama Iqbal Medical College, University of Health sciences, Lahore, Pakistan

## Abstract

Hepatitis C virus (HCV) is a major cause of chronic liver diseases including steatosis, cirrhosis and hepatocellular carcinoma. Currently, there is no vaccine available for prevention of HCV infection due to high degree of strain variation. The current treatment of care, Pegylated interferon α in combination with ribavirin is costly, has significant side effects and fails to cure about half of all infections. The development of *in-vitro *models such as HCV infection system, HCV sub-genomic replicon, HCV producing pseudoparticles (HCVpp) and infectious HCV virion provide an important tool to develop new antiviral drugs of different targets against HCV. These models also play an important role to study virus lifecycle such as virus entry, endocytosis, replication, release and HCV induced pathogenesis. This review summarizes the most important *in-vitro *models currently used to study future HCV research as well as drug design.

## Introduction

HCV infection is a serious global health problem that affects 180 million people worldwide and 10 million people in Pakistan [1]. It is estimated that three to four million people are infected with HCV every year. HCV causes acute and chronic hepatitis which can eventually lead to permanent liver damage and hepatocellular carcinoma [2]. Of those acutely infected with HCV, around 85% develop chronic infection. Approximately 70% of patients with chronic viremia develop chronic liver disease, 10-20% of which develop liver cirrhosis. Hundreds of thousands of people die each year from liver failure and liver cancer caused by this disease.

**Figure 1:**
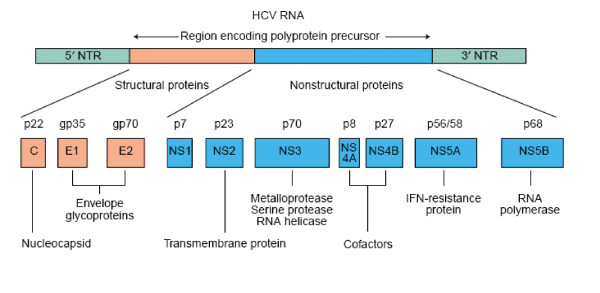
**Proteins encoded by the HCV genome**. HCV is formed by an enveloped particle harbouring a plus-strand RNA of ~9.6 kb. The genome carries a long openreading frame (ORF) encoding a polyprotein precursor of 3010 amino acids. Translation of the HCV ORF is directed via a 340 nucleotide long 5' nontranslated region (NTR) functioning as an internal ribosome entry site; it permits the direct binding of ribosomes in close proximity to the start codon of the ORF. The HCV polyprotein is cleaved co- and post-translationally by cellular and viral proteases into ten different products, with the structural proteins (core (C), E1 and E2) located in the N-terminal third and the nonstructural (NS2-5) replicative proteins in the remainder. Putative functions of the cleavage products are shown [4]

HCV is a small enveloped virus with a positive sense, single-stranded RNA genome that encodes a large polyprotein of 3010 amino acids. The polyprotein is co- and posttranslationally processed by cellular and virally encoded proteases to produce four structural (Core, E1, E2 and P7) and six non-structural (NS2, NS3, NS4A, NS4B, NS5A, NS5B) proteins [3,4]. Among the structural protein, HCV envelop protein E1 and E2 are highly glycosylated and play an important part in cell entry. HCV NS3 serine protease and NS5b play an important role in replication. HCV NS3 serine protease, NS5B RNA-dependent RNA polymerase and HCV structural proteins are important targets for antiviral drug development (Figure 1).

On the basis of nucleotide variation HCV is divided into six major genotypes and more than 80 subtypes. There is 30-50% variation among viral genotypes and 15-30% among different subtypes while there is 1-5% variation in nucleotide sequence from a single HCV infected patient [5,6]. They occur in different proportion in different parts of the world. Genotype 1a and 1b are the most common genotypes in the United States and Europe [7,8]. The most prevalent HCV genotype in Pakistan is 3a followed by 3b and 1a [9].

Presently, there is no vaccine available for prevention of HCV infection due to high degree of strain variation. Current therapeutic options for hepatitis C are limited, especially for genotype 1. For genotypes 2 and 3, pegylated interferon in combination with ribavirin, can lead to a sustained virological response in up to 80% of patients [10]. However, the therapy is expensive and often associated with side effects that may lead to discontinuation of therapy [11]. Hemolytic anemia, cough, shortness of breath & treatogenicity are the most common adverse effect associated with ribavirin treatment, and muscle aches, fatigue & neuropsychiatric adverse effects of IFN-α lead to premature cessation of therapy in 10 to 20% of patients [12,13]. Moreover, cost of interferon for 6 month treatment ranging from PKR 50,000 to 150,000 is beyond the financial range of most patients. Hence, there is need to develop anti HCV agents, both from herbal sources and synthetic chemistry which are less toxic, more efficacious and cost-effective. In this review, we summarizes the most important *in-vitro *models currently used to study future HCV research as well as drug design.

## HCV Infection System

In the past, research into HCV has been hampered due to lack of a reliable cell culture system. Conventional virological methods failed to initiate productive HCV infection. With the passage of time, new molecular techniques made it possible to develop an efficient *in-vitro *culture system to facilitate the study of HCV. Initial attempts to established HCV infection used primary cells from humans and chimpanzees. Primary human foetal hepatocytes infected with HCV-containing sera detected the positive strand of virus but the replication is low [14]. Human fetal hepatocytes supported HCV replication after infection with patient sera of 1a, 1b, 2a, 2b, and 3 HCV genotypes. HCV infected hepatocytes released HCV into medium for at least 2 month and HCV core protein and HCV negative-strand RNA also detected in infective cells. Viral replication had some cytotoxic effects on the cells due to production of interferon as a component of the antiviral response [15].

Due to short passage life and contamination problems in primary hepatocytes, scientists tried to develop immortalized human hepatoma cell lines. Many cell lines supported HCV infection and replication *in-vitro *such as Human T-lymphocyte cell lines, human fibroblast cells (VH3), peripheral blood mononuclear cells (PBMCs) and hepatocytes. But, hepatocytes are the target cells for HCV replication. Hepatoma cell line 7721 is susceptible to HCV by incubation of cells with HCV infected serum. HCV RNA is detected for at least three months following infection. This result also suggested that if the HCV-infected hepatoma cells were co culture with PBMCs, they were able to transfer the virus into PBMCs [16].

A human hepatocyte cell line, PH5CH, which is immortalised with simian virus 40 large antigen, was extensively studied. Although found to be more susceptible to HCV infection than others, the system was still inefficient [17]. Studies looking at hepatoma cell lines HepG2 and HuH-7 gave poor results even though conditions were changed extensively to try to optimise the approach [18,19]. Mizutani looked at infection of the human T-cell line, MT-2, which harbours human T-cell leukaemia virus-1 (HTLV-1). Although susceptible to HCV infection, it was not possible to produce long-term infection. Infection of Daudi cells, a B-lymphoplastoid cell line, managed to produce long-term infection for up to 1 year [20] but addition of the virus led to cellular cytopathic affects. It was possible to infect a chimpanzee with supernatant obtained after 58 days of culturing in Daudi cells, but infectivity was low. Other attempts were made to culture virus directly from cells of infected liver biopsies from persistently infected patients [21]. However, replication efficiency was low and reproducibility of the system was poor.

## HCV sub-genomic replicon

**Figure 2:**
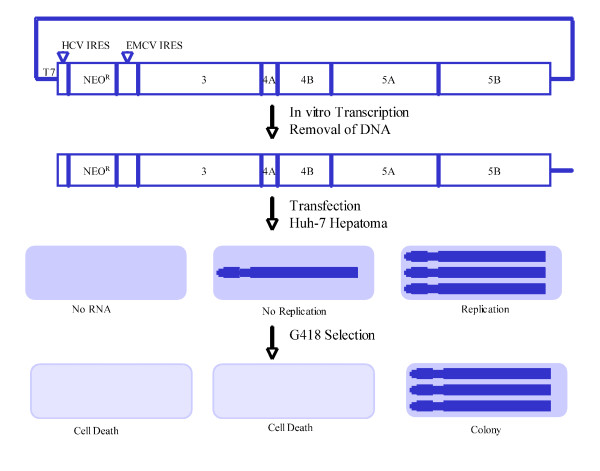
**The HCV subgenomic replicon system**.

The HCV replicon system replicates a modified HCV genome to high levels in human hepatoma (Huh-7) cells [22,23]. Replicons are either subgenomic (containing only the non-structural proteins for RNA replication) or genomic in length (contains the entire HCV genome). Both types of replicons contain the neomycin phosphotransferase gene for selection. A bi-cistronic replicon was created with the inclusion of an encephalomyocarditis virus (EMCV) IRES before the HCV non-structural genes. All genes are driven by a T7 promoter. Following transcription with T7 RNA polymerase, replicon RNA is transfected into Huh-7 human hepatoma cells. RNA replication allows cells to grow and form colonies in the presence of the antibiotic G418 (Figure 2).

Some Replicons containing a luciferase gene to replace the neomycin phosphotransferase gene were used in transient assays to identify adaptive mutations. The replicon acquired adaptive mutations by unknown mechanism. Adaptive mutations are generally detected in NS3, NS5A and NS5B proteins [22,24]. Adaptive mutation in NS5A region causes interferon resistance. The highly adapted 5.1 replicon contained three adaptive mutations (two in NS3 and one in NS5A). These adaptive mutation strongly increase RNA replication [25].

A full-length clone bearing three adaptive mutations was not infectious to chimpanzees, while a clone bearing one adaptive mutation could infect and this mutation reverted back to wild-type [26]. Cellular factors were also important for replicon establishment in cell culture. Replicons of passage number 128 replicated 100-fold more efficiently than those in cells at passage number 15 [24]. Characterization of cells that harbored replicons showed that they were able to maintain autonomously replicating RNA for over one year. Furthermore, viral RNA was still detectable 10 months after removal of selection by neomycin. Replicon-bearing cells showed no obvious signs of cytopathogenicity. Viral proteins were localized to ER membranes and replication and expression were linked to the cell cycle [27]. Long term treatment of replicon-harboring cells with IFN-α effectively removed or "cured" cells of the replicon [28]. Sub-genomic replicons, have been extremely useful for the screening of chemical libraries for novel molecules with antiviral actions against HCV.

## Infectious HCV virion

Recent studies have led to the development of infectious HCV culture systems. Wakita and his colleague developed genotype 2a full length replicon (JFH-1) which was isolated from a Japanese patient with fulminant hepatitis. This HCV full length genome replicates efficiently and produce virus particle (HCVcc) in Huh- 7 [29]. Chimaeric constructs of JFH-1 with the structural region of the J6 genotype 2a clone improved the infectivity [30].

Further refinements have led to the development of Huh7 derived cell lines (Huh7.5.1) which result in increase the viral titer 10^4 ^- 10^5 ^infectious units per ml of culture supernatant and these cell lines are highly permissive to JFH-1 virus infection [31]. JFH-1 infectious particle is an ideal tool to study all aspects of the HCV life cycle including viral attachment, entry, trafficking, replication. Virus particles can be neutralized with CD81 antibody and monoclonal antibody against the viral glycoprotein E1 and E2 [32]. HCVcc replication can also be inhibited by interferon-alpha and by several HCV-specific antiviral compounds, suggesting JFH-1 infectious culture system, is a powerful tool to study antiviral drugs and vaccines. But, there are some limitation to use JFHI infectious particles such as these particles isolated from Fulminent Hepatitis which is rare event in hepatitis. Another important limitation that HCV particles is based on genotype 2 which is not dominant genotype in world and Pakistan.

## HCV producing Pseudo particle (HCVpp)

**Figure 3:**
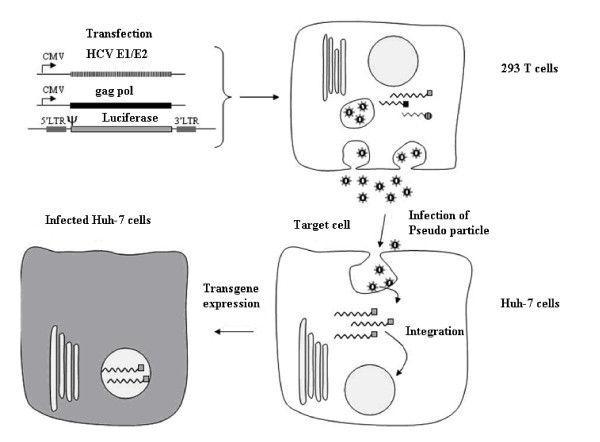
**Scheme of HCVpp production and infection**. HEK-293T cells were transfected with CMV-Gag-Pol, Luciferase vector and HCV GP expression constructs. Successfully transfected 293T cells assemble HCVpp intracellularly and secrete them into the supernatant. The HCVpp-containing supernatant can be harvested. The supernatant can then either be used unmodified or conditioned (centrifugation, drug treatement etc.) to infect target cells. HCVpp attach to the target cells, become endocytosed and fuse with the endocytic membranes to release the retroviral core containing the luciferase vector into the cytoplasm. The Luciferase vector is then reverse transcribed and integrated into the host-cell genome. 24 to 96 hours after infection, transgene expression can be analyzed.

HCV pseudotype particles were produced to study the early stages of viral life cycle. HCVpp were produced by transfecting the three vectors in Human embryo kidney cells (293T). The first vector encodes retroviral Gag and Pol proteins which are responsible for particle budding at the plasma membrane and RNA encapsidation. The second vector encodes a reporter protein (Luciferase) or GFP. The third vector encodes HCV glycoproteins E1 and E2, which are necessary for viral tropism and fusion of HCV pseudo type particles with target cell membrane. 293T cells secreted virus pseudo particle an average 10^5 ^particles/ml, which can be used to infect Huh 7 cells and infectivity is eveluated by quantification of amount of luciferase or GFP expressed in Huh-7 cells. These virus like particles can be neutralized with monoclonal antibody against the viral glycoprotein E1, E2 and sera of HCV infected patient [32,33] and are a powerful tool to identify inhibitors which block HCV entry. HCVpp are also essential to find out fusion mechanism of virus (Figure 3).

## Conclusion

HCV infection is a serious global health problem necessitating effective treatment. Currently, there is no vaccine available for prevention of HCV infection due to high degree of strain variation. The current treatment of standard, Pegylated interferon α in combination with ribavirin is costly, has significant side effects and fails to cure about half of all infections. The development of robust, cell-based replication and entry systems such as HCV sub genomic replicons, HCVpp and Cell culture producing virion will certainly accelerate the anti-HCV drug development process. With determination and innovation, we will undoubtedly meet the medical needs of those chronically infected with HCV.

## Competing interests

The authors declare that they have no competing interests.

## Authors' contributions

UAA, SNK, ZN, SRD contributed equally in manuscript write up. All the authors read and approved the final manuscript.
